# Variant fatty acid-like molecules Conjugation, novel approaches for extending the stability of therapeutic peptides

**DOI:** 10.1038/srep18039

**Published:** 2015-12-11

**Authors:** Ying Li, Yuli Wang, Qunchao Wei, Xuemin Zheng, Lida Tang, Dexin Kong, Min Gong

**Affiliations:** 1 Tianjin Neurological Institute, Tianjin Medical University General Hospital, China; 2 Tianjin Institute of Pharmaceutical Research, China; 3 Department of Pharmacy, Tianjin Medical University, Tianjin, China; 4 Department of Oncology, University of Oxford, UK

## Abstract

The multiple physiological properties of glucagon-like peptide-1 (GLP-1) make it a promising drug candidate for the treatment of type 2 diabetes. However, the *in vivo* half-life of GLP-1 is short due to rapid degradation by dipeptidyl peptidase-IV (DPP-IV) and renal clearance. The poor stability of GLP-1 has significantly limited its clinical utility; however, many studies are focused on extending its stability. Fatty acid conjugation is a traditional approach for extending the stability of therapeutic peptides because of the high binding affinity of human serum albumin for fatty acids. However, the conjugate requires a complex synthetic approach, usually involving Lys and occasionally involving a linker. In the current study, we conjugated the GLP-1 molecule with fatty acid derivatives to simplify the synthesis steps. Human serum albumin binding assays indicated that the retained carboxyl groups of the fatty acids helped maintain a tight affinity to HSA. The conjugation of fatty acid-like molecules improved the stability and increased the binding affinity of GLP-1 to HSA. The use of fatty acid-like molecules as conjugating components allowed variant conjugation positions and freed carboxyl groups for other potential uses. This may be a novel, long-acting strategy for the development of therapeutic peptides.

Since the observation of free fatty acids in 1949, it was observed that free fatty acids were capable of binding with human serum albumin (HSA)^1^. HSA, the most abundant protein in blood, is an important protein involved in drug metabolism^2^. Most administered drugs are bound to HSA and are dissociated in blood circulation and tissue transfer^3^. The special function of binding to HSA makes fatty acids a potent avenue to extend the blood retention time for drugs with poor stability. Many studies have affirmed that conjugating fatty acids with therapeutic agents (*e.g.,* protein, peptides and siRNA) could delay the absorption rate, prolong the duration of the circulation and protect against proteolysis^4^,^5^,^6^,^7^. Interestingly, binding to HSA seemed to improve stability and was especially suitable for unhealthy subjects. The physiological environment of the human body induced the binding of fatty acids to HSA^8^. For example, only 1–2 fatty acid molecules bind to one HSA molecule under normal conditions. However, the number of bound fatty acids increased to 6–7 molecules per HSA molecule in diabetic subjects^9^. This suggested that fatty acid-conjugating drugs could exhibit enhanced sustained release profiles for diabetic subjects compared with those for healthy subjects. There are several fatty acid-conjugating drugs approved by the FDA for various clinical indications, including insulin detemir and Liraglutide. In insulin detemir, myristic acid was conjugated with a lysine side-chain in the α-chain of insulin to achieve the sufficient stability to meet the clinical requirements for treating T2DM^10^,^11^. The conjugation of the C-16 fatty acid resulted in a significant increase in the therapeutic half-life (13 h vs. 5 min)^12^,^13^. Fatty acid conjugation has also been developed as a popular strategy to extend the stability of therapeutic agents having poor stability.

The conjugation of fatty acids with active therapeutic agents requires complex steps, which unfortunately contributes to a extremely challenging synthesis. In native fatty acids, the carboxyl group is the only active site for conjugation, which indicated that a basic group in the active drug is necessary. However, due to the structural conformations and receptor binding properties of active drugs, the potent optimal conjugating position for fatty acids lacks basic groups. To overcome this obstacle, an active basic group was introduced through molecular modification or a linker was employed to conjugate drugs and fatty acids together. Accordingly, we presumed that a modified fatty acid analog could provide an optimized conjugating strategy for fatty acids. To simplify the synthesis, fatty amines were conjugated to peptide carboxyl groups, such as Asp or Glu residues. Fatty acid-like molecules were selected because they contained both active amino and carboxyl groups.

The aim of the current study is to extend the stability of glucagon-like peptide-1 (GLP-1) by conjugating fatty acid analogs. GLP-1 is a gut hormone released from intestinal L cells following oral glucose administration. It is considered a potent therapeutic strategy for T2DM because GLP-1 is vital for insulin secretion in a blood glucose level-dependent manner^14^,^16^. The use of GLP-1 avoids the risk of hypoglycemia, which provides a distinct clinical utility, unlike insulin. Furthermore, GLP-1 promotes β-cell regeneration, making it a possible treatment for type 1 diabetes^17^,^18^. It was also confirmed that GLP-1 inhibits apoptosis (programmed cell death) in β-cells; this suggests that GLP-1 may be an effective agent for the treatment of T1DM^19^. However, the poor stability of native GLP-1 (3–5 min) has significantly limited its clinical utility due to the rapid degradation catalyzed by the enzyme dipeptidyl peptidase IV (DPP-IV). The extremely poor stability renders the therapeutic administration of GLP-1 impractical; thus, many efforts have focused on altering the pharmacokinetic properties of GLP-1 by developing a series of derivatives and analogs^20^. As introduced earlier, Liraglutide is a long acting GLP-1 derivative containing two modifications: a substitution of Arg^21^ for Lys^21^ and the C-16 free fatty acid derivative attachment via a spacer to Lys^22^,^23^,^24^. The free-fatty acid promotes the binding between Liraglutide and albumin^25^; accordingly, the absorption rate of Liraglutide is delayed at the injection site, and its clearance rate is decreased^25^,^26^.

Because fatty acids have excellent physicochemical characteristics, it is worthwhile to evaluate the efficiency of a modified fatty acid in a drug conjugate. Herein, several fatty acid-like molecules were employed and conjugated to GLP-1 peptides to prolong the *in vivo* half-life. These novel fatty acid-like molecules and GLP-1 conjugates were then analyzed for their receptor binding capacities, stability, and glucoregulatory and long-lasting anti-diabetic effects in animal models.

## Materials and Methods

### Materials

DPP IV enzyme (0.1 mg/ml; ~95% purity) and human serum albumin were purchased from Sigma-Aldrich. Human GLP-1 (7–37) ELISA kits were purchased from Millipore, Inc., and cAMP kits were purchased from CisBio Bioassays. The rat INS-1 cell line was obtained from Ying Li of Tianjin Medical University General Hospital. A rat insulin detection kit was purchased from Phoenix Technology, Inc. A one–touch blood glucose meter and filters were purchased from Abbott. Other chemicals were purchased from Sigma unless otherwise specified.

### Animals

Kunming mice, male ZDF (fa/fa) rats, lean male ZDF rats, and male Wistar rats were purchased from Shanghai Laboratory Animal Co. (SLAC), China Academy of Sciences (Shanghai, China).

### Ethics statement

All studies in animals were performed in accordance with the approved guideline in Animal Experiments Inspectorate, China. All experimental protocols were approved by Tianjin Institute of Pharmaceutical Research committee.

### Peptide Synthesis

Peptides were ordered from the Peptide Center of Wuxi AppTec Company (Shanghai, China, Contract No. 216548). Peptides were synthesized using solid-phase peptide synthesis (Liberty 1, CEM). The synthesized peptides were purified by a Surveyor HPLC system through a C18 analytical column; the UV detection wavelength was set at 220 nm. The column was eluted at a flow rate of 0.5 ml/min in H_2_O containing 40% acetonitrile and 0.1% trifluoroacetic acid. HPLC analyses were performed at ambient temperature following lyophilization. The freeze-dried peptides were weighed and dissolved in saline to make 1 mg/ml stock solutions for further analyses.

### GLP-1 receptor binding assay

The binding affinities of the wild-type and derivatives to the GLP-1 receptor (GLP-1R) were determined by cellular ELISA assay. INS-1 cells, which overexpress the GLP-1 receptor, were seeded in 96-well plates (BD Biosciences) at approximately 95% confluence. Cells were rinsed with PBS and fixed with 4% paraformaldehyde (Thermo Scientific) for 10 min at room temperature and quenched for 5 min with 2% glycine in PBS, pH 7.5. For the binding capacity experiment (n = 4), cells (1 × 10^5^) were incubated with derivatives at various concentrations (10 pM—10 μM) and wild-type GLP-1. After a 2 h incubation at 37 °C in a final volume of 100 μl, the excess peptides were washed, and the cells were blocked with 5% BSA (BD Biosciences). Bound residual peptides and wild-type GLP-1 were detected by the GLP-1 (7–37) ELISA kit. Colorimetric changes were analyzed by reading the absorbance at 450 nm in a SpectraMax M5 (Molecular Device) microplate reader. The peptide concentration was plotted against UV absorbance, and the Kd was calculated using Origin 7.0 software.

### Stability of GLP-1 and fatty acid conjugates

The measurement of the stability of the GLP-1 derivatives is fundamental to estimate the improved properties of long-acting GLP-1 derivatives. To determine whether the GLP-1 derivatives were protected against the DPP IV enzyme, the derivatives were incubated in DPP IV enzymatic solution with or without the presence of HSA. DPP IV enzyme (0.1 mg/ml) was dissolved in 25 mM potassium phosphate buffer containing 25 mM potassium chloride and 5 mM magnesium chloride, pH 7.0. Wild-type GLP-1, or each derivative (1 mg/ml), was added into the solution for a final HSA concentration of 2%. The mixtures were incubated at 37 °C for 24 hours, and full-length GLP-1 peptides were quantified using the GLP-1 (7–37) ELISA kit at 0, 0.1, 0.5, 2, 4, 8, 12 and 24 h after incubation.

### Blood retention time measurements

Wild-type GLP-1 and derivatives (compounds 18, 21, 22, 23 and 24) were injected (500 μg/kg body weight) subcutaneously into male Wistar rats (n = 5 per group). Blood samples (200 μl) were subsequently obtained from the treated and control groups (i.e., rats treated with only saline) at 0.5, 1, 4, 12, 24, 48 and 96 h. Serum samples were analyzed using the GLP-1 (7–37) ELISA kits.

### Insulin stimulation assay

After observing the retained receptor binding affinity and improved stability by several GLP-1 long-acting derivatives, i.e., compounds 18, 21, 22, 23 and 24 (highlighted in Table 1), the insulin secretion assays were carried out by injecting those derivatives into male Wistar rats (n = 6 per group). Wild-type GLP-1 (100 μg/kg body weight) and saline were injected subcutaneously as controls. Glucose (10 g/kg body weight, a standard dose for oral glucose administration) was orally administered 30 min after peptides injections. Blood samples were collected by tail vein incision at 5, 15, 30, 45, 60, 90 and 120 min after glucose administration. Blood samples were then assayed for insulin levels using a Rat Insulin RIA kit.

### cAMP accumulation measurement

For the cAMP assay, INS-1 cells (1.0 × 10^5^ cells) were seeded and plated in each well of a 96-well opaque white plate. After 24 h, the media was replaced with RPMI 1640 medium containing 500 μM 3-isobutyl-1-methylxanthine (IBMX, an inhibitor of cAMP phosphodiesterase). Subsequently, 20 μg of either GLP-1 or each derivative was titrated. The assay plate was titrated with 2.5 μl/well cAMP and an equal volume of anti-cAMP conjugate (Cisbio) after a 1 h incubation. The homogenous time-resolved fluorescence (HTRF) signal was read on the SpectraMax M5 (Molecular Device) microplate reader after 60 min. The ratio of the absorbances at 665 nm and 620 nm (×10,000) was calculated and plotted.

### Glucoregulatory assay

To clarify whether compounds 22 and 24 possessed glucose regulatory activity, single dose glucoregulatory assays were performed. In this assay, the derivatives were administered once, and the blood glucose levels were monitored over 120 h. It was presumed that the apparent half-lives of the derivatives could be obtained by this single-dose glucose tolerance test, which would be beneficial for the determination of the administration frequency in future long-term glucose tolerance tests. Each derivative (300 μg/kg body weight) was subcutaneously injected into fasting male Wistar rats (n = 7 per group) 30 min prior to glucose administration. GLP-1 and saline were injected into the control animals. Rats were given 2 g glucose/kg body weight via intraperitoneal injections. Blood was drawn from the tail vein, and glucose levels were measured using a glucometer 30 min after glucose administration. Chronic glucose injections (2 g/kg body weight) were administered 30 min prior to each blood glucose measurement time point during the 120-h experimental period. After the observations of the prolonged glucose clearance activity of compounds 22 and 24, the dosage-effect relationship was also investigated for these derivatives in 48-h experiment periods at dosages of 10, 50, 250, and 1250 μg/kg body weight, respectively.

### Potent anti-diabetic activity of compound 22 and 24

According to the prolonged, long-acting properties showed by compounds 22 and 24, the long-term glucose tolerance in ZDF rats was investigated to determine the anti-diabetic activity of the derivatives. Male ZDF rats (n = 10 per group) were treated with compounds 22 or 24 (100 μg/kg/2 days) for the entire experimental period (42 days). The control groups were injected with wild-type GLP-1 and Liraglutide (100 μg/2 days). HbA_1c_ levels were assessed using a DCA 200 analyzer (Bayer Diagnostics), and the body weight and glucose levels were monitored during the experimental period.

### Toxicity test of compound 24

Wistar rats (n = 8) and Kunming mice (n = 30) were employed in this study to investigate the acute toxicity of compound 24. Compound 24 was intraperitoneally administered with three doses of 5, 15 and 50 mg/kg/2 days for 30 days. Controls received a similar volume of sodium dihydrogen phosphate. At the end of the experimental period, overnight fasted animals were sacrificed by cervical decapitation under light ether anesthesia and blood was collected, serum was separated by centrifuging at 3,000 rpm for 10 min. The serum was used for the assay of the biochemical parameters using Chemistry Analyzer SELECTRAE (Vital, Netherlands).

### Statistical analyses

Student’s *t*–test was used to analyze the data. Unless otherwise stated, the results are reported as the mean ± standard error. P values less than 0.05 were considered significant.

## Results

### Measurement of receptor binding affinity

There are two main approaches for the measurement of the binding affinity of GLP-1 to GLP-1 receptor, radiolabelled GLP-1 RIA and native GLP-1 ELISA. Compared to radiolabelled GLP-1 approach, GLP-1 ELISA approach is a cheaper and convenient procedure but possesses main obstacle. The recognition and binding of GLP-1 to antibody is crucial for GLP-1 ELISA, which means the structural or conformational alteration upon GLP-1 molecules, such as GLP-1 derivatives in this study, might attenuate this binding affinity. In our research, the GLP-1 ELISA kit was employed initially to ascertain whether this approach was suitable for our GLP-1 derivatives. Fortunately, the structural modification of GLP-1 derivatives in this study remained the binding activity to antibody in ELISA kit, which means the receptor binding assay is capable to conducted using GLP-1 ELISA approach.

Using a cellular ELISA-based receptor binding assay, the binding capacities of wild-type GLP-1 and derivatives to GLP-1 receptor were determined in INS-1 cells. The results showed that while some derivatives retained similar binding capacities to that of wild-type GLP-1, others did not (i.e., compounds 1–3 and 7–12) (Table 1). The results suggested that the conjugation positions may alter the structural conformations of the protein, which might affect the receptor binding characteristics.

### Stabilization assays

**Figure 1:**
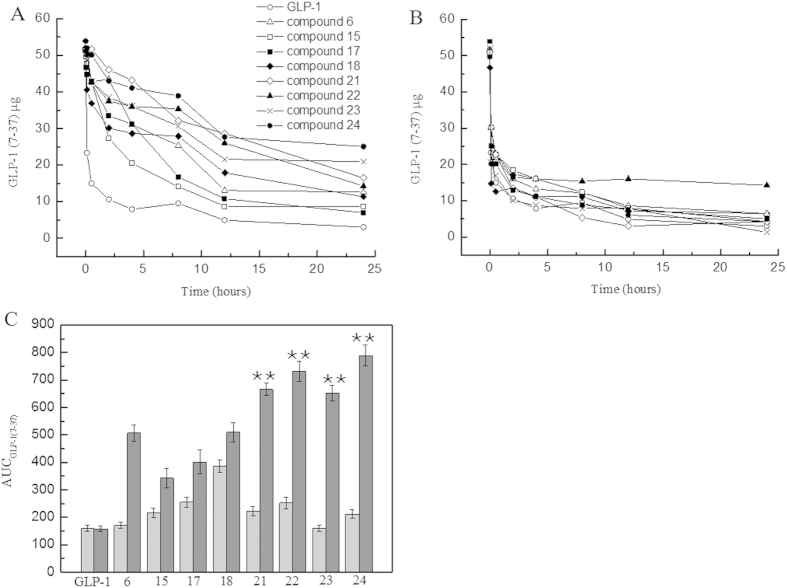
Stabilization studies of GLP-1 derivatives against DPP IV proteolysis. Panel (**A**) A pharmacokinetic study of GLP-1 (○) and derivatives in DPP IV protease solution. Panel (**B)** A pharmacokinetic study of GLP-1 (○) and derivatives in DPP IV protease solution in the presence of human serum albumin. **Legend:** The results indicate that the compound 6, 15, 17, 18, 21, 22, 23 and 24 was still detectable at 24 h, whereas native GLP-1 was rapidly degraded within 5 min, showing that these derivatives have prolonged stabilities upon the binding to human serum albumin. Panel (**C**) AUC_GLP-1(7–37)_ of the circulating GLP-1 concentration in proteolysis solution. **Legend:** The calculated AUC_GLP-1(7-37)_ shows that the amount of compound 21, 22, 23 and 24 was significantly increased compared to native GLP-1, P < 0.01. **Conditions:** GLP-1 and fifteen GLP-1 derivatives were incubated in DPP IV solution with or without the existence of human serum albumin. A human GLP-1 (7–37) ELISA kit was used to quantitatively determine the half-life of GLP-1 and its derivatives in human serum, according to the manufacturer’s instructions.

The derivatives observed to have binding affinity to the receptors were subjected to a proteolysis assay by incubating with DPP IV enzyme in order to investigate whether these derivatives were more resistant to DPP IV protease than wild-type GLP-1 upon the presence of human serum albumin. Within 24 h, the derivatives 6, 15, 17, 18 and 21–24 exhibited slower degradation rates than that of the wild-type GLP-1, as shown in Fig. 1A. The wild-type GLP-1 degraded rapidly in proteolysis buffer, and showed the same degradation profile in a solution containing HSA. When the stabilities of the derivatives in the proteolysis buffer without HSA were compared, the results indicated that the improved stabilities were attributable to the presence of human serum albumin. The data also suggested that the stabilities were dependent on the length of the carbon chain in fatty acids. The C-16 fatty acid conjugates resulted in longer half-lives than those of the C-12 and C-8 conjugates. The calculated AUC result indicated that the detectable amount of the GLP-1 derivatives significantly increased compared with wild-type GLP-1, as shown in Fig. 1C. From the calculated AUC_0–24 h_, the data indicated that the amount of undigested GLP-1 fragments from compounds 22 and 24 were approximately 3.5-fold greater compared with that of the wild-type GLP-1 (P < 0.01). In summary, the results clarified that conjugated fatty acid molecules are beneficial for extending the stability of GLP-1 against DPP IV enzymes.

### Blood retention time measurement

**Figure 2:**
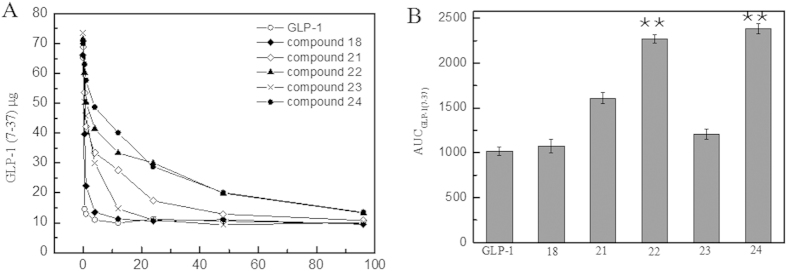
Blood retention time measurement of GLP-1 derivatives in Wistar rats. Panel (**A**) A pharmacokinetic study of the GLP-1 derivatives in Wistar rats. Panel (**B**) AUC _GLP-1 (7-37)_ of the circulating GLP-1 concentration in treated Wistar rats. **Legend:** The results show that the serum level of native GLP-1 (○) was rapidly decreased by proteolysis and that the compound 22 (▲) and compound 24 (●) derivatives extended the stabilities *in vivo* compared with native GLP-1. In addition, the AUC data indicated that a 2.6- and 2.7-fold increase of circulating amount of compound 22 and 24, respectively. **Conditions:** Wistar rats were injected with the GLP-1 derivatives (compound 18, 21, 22, 23 and 24) (500 μg/kg body weight, n = 5). Blood samples were taken from the tail vein at various time points, and the peptide levels were measured using a GLP-1 (7–37) ELISA kit.

Figure 2 shows that after the injection into the Wistar rats, the wild-type GLP-1 rapidly degraded to baseline levels (10 μg) at 0.5 h. Compared with the rapid degradation of the wild-type GLP-1, the GLP-1 derivatives (compounds 18 and 21–24) exhibited improved blood retention behaviors. The results indicated that compounds 22 and 24 might be the optimal mutations derived from wild-type GLP-1 because of their retained binding capacities and extended half-lives. Accordingly, they were employed in further assays to investigate their biological activities, such as in insulin secretion, cAMP accumulation, blood glucose regulation and long-acting anti-diabetic activity.

### Effects on insulin secretion and cAMP accumulation

**Figure 3:**
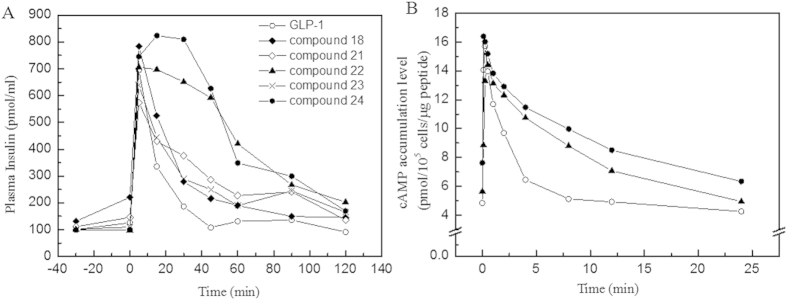
Effects of GLP-1 derivatives on the insulin stimulation and cAMP accumulation after oral glucose administration. Panel (**A**) The stimulation of insulin secretion by native GLP-1 (○) and five derivatives (compound 18, 21, 22, 23 and 24) in Wistar rats after oral glucose administration. Panel (**B**) The effect of GLP-1 derivatives on cAMP levels in INS-1 cells. **Legend:** The results indicate that the oral administration of glucose increased insulin levels in the rats. The levels of secreted insulin induced by GLP-1 arrived the peak at 10–20 min and returned to baseline at 40 min. The insulin levels in Wistar rats treated with compound 22 or 24 showed distinct difference in insulin secretion. Compared with GLP-1, these two derivatives stimulated the prolonged secretary response significantly. In similarity, the cellular cAMP level stimulated by GLP-1 peaked to 16.34 ± 1.04 pmol/10^5^ cells in 2 min before declining to baseline at 10 min. However, the cells treated with compound 22 (▲) or 24 (●) exhibited longer response than cells treated with GLP-1 remarkably. **Conditions:** GLP-1 and derivatives (100 μg GLP-1/kg body weight) were injected into Wistar rats; glucose was administered orally (10 g/kg). The concentration of insulin was measured using a rat insulin detection kit. Total cellular cAMP was measured in INS-1 cells (1.0 × 10^5^) at the indicated times using an HTRF-cAMP kit. The data are presented as the means ± SE, P < 0.01.

Next, we determined whether the biological functions of compounds 22 and 24 were retained upon the conjugation of the fatty acid molecules. Increased insulin secretion activity, as induced by oral glucose administration, is a characteristic trait of GLP-1; the presence of this activity is a crucial indicator in the evaluation of the success of the GLP-1 derivatives. In the control group treated with only wild-type GLP-1, the administration of glucose dramatically increased the insulin level to 694.38 ± 28.65 pmol/L at 10 min (phase I), which returned to baseline levels (108.02 ± 15.46 pmol/L) by 45 min (phase II). For derivative compounds 22 and 24, the modification of GLP-1 did not alter this native physiological function. Both GLP-1 derivatives showed similar spectral feature in which the insulin secretion levels peaked to similar levels at 20 min and maintained prolonged stimulations compared with that of the wild-type GLP-1 (Fig. 3A). In addition, the cAMP accumulation assay demonstrated increased cAMP levels induced by compounds 22 and 24 upon glucose administration (Fig. 3B).

### Half-life measurement of compounds 22 and 24

**Figure 4:**
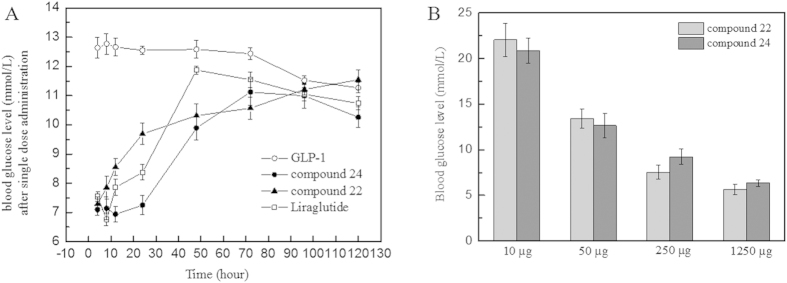
Effects of compound 22 and 24 on glucose tolerance after a single-dose injection. Panel **(A**) The effect of a single injection of the GLP-1 derivatives and Liraglutide on glucose regulation in Wistar rats. Panel (**B**) The dosage-efficacy of compound 22 and 24, 24 h after administrations. **Legend:** The administration of compound 24 maintained the blood glucose concentration at normal levels for 48 hours after a single-dose injection approximately, suggesting improved blood glucose clearance activity than liraglutide in long term manner. **Conditions:** Fasting Wistar rats were injected with the GLP-1 derivatives or GLP-1. Glucose (2 g/kg body weight) was administered 30 min before each time point (0–120 h).

To examine the blood glucose clearance activity of compounds 22 and 24, glucose tolerance tests (GTTs) were performed 120 h after the single-dose administration of GLP-1 mutants, wild-type GLP-1 and Liraglutide into male Sprague-Dawley rats (n = 6 per group). The peptides were injected 1 h before the first measuring point, and glucose was administered 30 min before the first measuring point. The rats treated with GLP-1 had a maximum blood glucose level of approximately 12 mmol/L over the whole experimental period of 120 h (Fig. 4). It was presumed that the GLP-1 had degraded before the first measuring point (1 h after GLP-1 administration). As predicted, the rats injected with compounds 22 or 24 showed better glucose tolerance in this single-dose injection experiment than those injected with wild-type GLP-1. Treatment with compound 22 resulted in blood glucose levels maintained at 7–9 mmol/L within 12 h and approximately at 48 h for compound 24. Compared with compound 24, the administration of Liraglutide failed to provide good control of blood glucose after 24 h. This result suggested the administration frequency of compound 24 could be reduced to one injection every two days.

### Long-term anti-diabetic activities of compounds 22 and 24

To assess whether the two derivatives had long–acting anti–diabetic effects, the glucose level, HbA_1c_ levels and body weight were monitored over 42 days. In this experiment, compound 22, compound 24, wild-type GLP-1 and Liraglutide were administered into ZDF rats (300 μg/kg body weight/2 days), and saline was administered to a blank group. The blood glucose level and body weight were monitored every 3 days. After 42 days of treatment, the HbA_lc_ levels decreased 1.09 ± 0.02% from 9.0 ± 0.11% (blank group), whereas treatment with GLP-1 alone failed to reduce the HbA_lc_ levels. Liraglutide only induced a 0.33 ± 0.12% decrease with this administration frequency. Combined with changes in the glucose level and the body weight index, the results clearly indicated that the compound 24 possessed longer–lasting anti–diabetic effects than that of Liraglutide; this was an improvement compared with the non-clinical data with Liraglutide administered at a frequency of one injection every two days^27^.

Finally, the acute toxicity and blood biochemistry tests for compound 24 were performed at the Research Center of New Drug Safety Evaluation in Tianjin according to the Good Laboratory Practice Guideline of China. Data suggested that no toxicity was observed, and compound 24 could be a safe, novel anti-diabetic drug candidate (see Tables in supplementary).

## Discussion

The ability of fatty acids to bind to human serum albumin has provided additional stabilization of potent drug candidates. The conjugation of fatty acid to ameliorate the poor stability of bioactive molecules has been a popular strategy for several decades due to the added efficiency and non-toxic effects^22^,^28^,^29^,^30^. Additional bioactive substances, such as proteins, peptides and nucleic acids, have recently exhibited excellent physiological characteristics and potent clinical utilities. However, their use in clinical practices has been significantly limited due to poor stabilities despite their excellent physiological advantages; an example of this is Glucagon Like Peptide-1 (GLP-1)^31^. The known physiological functions of GLP-1 implied that it plays a critical role in the regulation of glucose homeostasis and suggests that it is a feasible candidate in the treatment of type 2 diabetes mellitus^32^,^33^. Despite its attractive anti-diabetic action, the therapeutic potential of using native GLP-1 is limited by its short lifetime (<2 min) *in vivo*. This *in vivo* clearance rate is primarily due to rapid enzymatic inactivation by DPP-IV^34^ and a renal clearance of less than 10 min^21^. To provide any clinical utility, therapeutic derivatives of human GLP-1 would need to have extended half-life properties. The conjugation of fatty acids on GLP-1 has led to the successful approval and marketing of Liraglutide in 2010. However, the clinical utility of Liraglutide is still limited by its half–life (~13 h). Daily injections of Liraglutide are not ideal for the treatment of type 2 diabetes for patients. Until now, many efforts have focused on altering the pharmacokinetic properties of GLP-1 by developing a series of derivatives and analogs^35^,^36^,^37^, including conjugations of GLP-1 with human albumin or IgG^38^,^39^.

The conjugation of fatty acids on peptides is difficult and complex. Fatty acids prefer to conjugate with a lysine side-chain, which suggested that Lys residues are not involved in receptor binding, otherwise an additional linker or amino acid replacement would be necessary for conjugation. The challenges in fatty acid conjugation processes prompted the consideration of replacing fatty acids with fatty acid-like molecules in order to better facilitate conjugating strategies. In this study, the fatty acid amine and amino-terminal fatty acids [14-aminocaptonic acid] were conjugated to GLP-1. In this modified strategy, the fatty acid amine could be conjugated with the Glu and Asp side-chains, and the amino-terminal of the fatty acid could react with Lys, Glu and Asp because it contains both amino- and carboxyl groups. The utility of the amino-terminal fatty acids may have extensive applications, such as participation in peptide synthesis as an unnatural amino acid due to its structural similarity with amino acids.

Twenty-four GLP-1 derivatives with fatty acid molecule conjugates were designed and synthesized. The sequences are listed in Table 1, and their physiological properties (i.e., receptor binding affinity, insulin stimulation activity and long term blood glucose clearance characterization) were investigated. The cellular receptor binding assay demonstrated that the compounds highlighted in Table 1 retained binding affinity for the receptor, while the non-highlighted compounds did not retain binding affinities. Conjugations at ^3^Glu and ^9^Asp resulted in the loss of binding affinity because the N-terminus of GLP-1 potentially binds to the receptor^40^,^41^.

**Figure 5:**
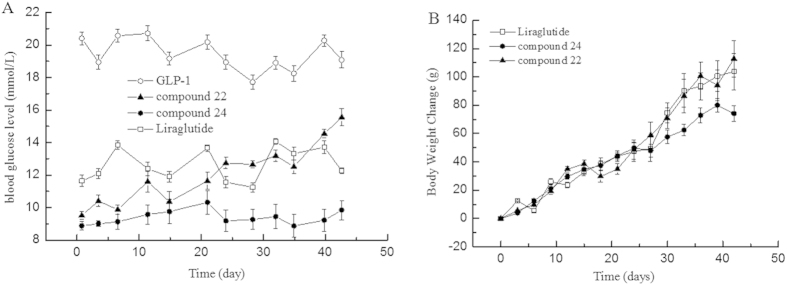
Effect of multiple doses of compound 24 over 42 days treatment. Panel (**A**) The long-lasting glucose regulatory effect of compound 24 (●) in ZDF rats. Panel (**B**) Body weight changes after the treatment of compound 24 in ZDF rats. **Legend:** The results indicate that the treated ZDF rats maintained relatively constant and lower glucose levels than the control rats; native GLP-1 failed to produce a similar glucose regulatory effect. Another compound 22 and liraglutide is not capable to provide satisfied blood glucose well-control. Treatment of compound 24 also lowered the body weight increase which is the physiological function of GLP-1. **Conditions:** Compound 24 (●) (300 μg /kg body weight) was administered every two days during the experimental period, which lasted 42 days. A glucometer was used to measure the glucose levels at various time intervals.

In the stabilization assay, it was clearly indicated that longer fatty acid carbon chains contributed to better stabilities, i.e., C16 fatty >C12 fatty >C8 fatty in Fig. 1. Moreover, the use of the amino-terminal fatty acids seemed to be beneficial for prolonging the stability of the derivatives than the use of fatty acids without a carboxyl group in same carbon chain length. Compounds 22 and 24 showed significantly lower degradation rates than those of compounds 6 and 21 against DPP IV, respectively. Data suggested that the existence of a free carboxyl group contributed to increasing the binding affinity to HSA; similar conclusions were reported by Spector^42^. To calculate the observed half-life of each derivative, a single dose glucose tolerance test was performed. Compound 24 exhibited remarkable blood glucose clearance efficacy; the observed half-life was calculated as 9.6 h. This half-life indicated that compound 24 could be administered as an anti-diabetic candidate every two days; the half-life of Liraglutide is 3.6 h in rats^43^. The insulin secretion assay demonstrated that these derivatives retained their insulin secretion activity (Fig. 3). The increased stabilities of derivative compounds 22 and 24 led to prolonged insulin stimulus responses, and the phase II insulin secretion was significantly increased in comparison with that produced by the wild-type GLP-1. Similarly, the cAMP levels and the calculated AUC indicated that the derivatives retained their physiological activity and improved the stimulus duration. The subsequent multi-dose glucose tolerance test confirmed that blood glucose was well-controlled with the administration of compound 24 every two days for 42 days. The HbA1c reduction caused by the administration of compound 24 was 1.09 ± 0.02%. The results suggested that compound 24 possessed better glucose regulatory action than Liraglutide. Like Liraglutide, the derivative compounds were capable of lowering the body weight for obese rats (Fig. 5).

Compared with Liraglutide, which exhibited a longer biological half-life (13 h) and fewer side effects (*e.g.,* nausea) than exenatide, compound 24 significantly increased the biological half-life of GLP-1 without disturbing the other physiological functions of GLP-1 (e.g., insulin secretion, cAMP accumulation, HbA1c reduction). The data from the rodent model indicated that the administration of the compounds every two days was capable of normalizing the blood glucose levels.

In conclusion, the findings in the current study provided an optimal strategy for producing long-acting peptides via fatty acid conjugation. This modified approach provided alternative means to conjugating peptides with fatty acid-like molecules. The conjugation of amino-terminal fatty acids is a powerful tool for improving the stability of clinical peptides because a free carboxyl group can increase the binding affinity to HSA.

## Additional Information

**How to cite this article**: Li, Y. *et al.* Variant fatty acid-like molecules Conjugation, novel approaches for extending the stability of therapeutic peptides. *Sci. Rep.* **5**, 18039; doi: 10.1038/srep18039 (2015).

## Supplementary Material

Supplementary Information

## Figures and Tables

**Table 1:** Sequences of GLP-1 derivatives and cellular binding constants to GLP-1 receptor.

PEPTIDE	Amino Acid Sequence	K_d_
GLP-1	^1^HAEGTFTSDVSSYLEGQAAKEFIAWLVKGRG^32^	14.25 ± 1.02 nM
compound 1	^1^HAEGTFTSDVSSYLEGQAA**K**(C8 acid)EFIAWLVKGRG^32^	36.21 ± 0.95 nM
compound 2	^1^HAEGTFTSDVSSYLEGQAA**K**(C12 acid)EFIAWLVKGRG^32^	39.86 ± 1.27 nM
compound 3	^1^HAEGTFTSDVSSYLEGQAA**K**(C16 acid)EFIAWLVKGRG^32^	33.76 ± 1.21 nM
compound 4	^1^HAEGTFTSDVSSYLEGQAAKEFIAWLV**K**(C8 acid)GRG^32^	15.41 ± 0.67 nM
compound 5	^1^HAEGTFTSDVSSYLEGQAAKEFIAWLV**K**(C12 acid)GRG^32^	14.32 ± 1.10 nM
compound 6	^1^HAEGTFTSDVSSYLEGQAAKEFIAWLV**K**(C16 acid)GRG^32^	13.71 ± 0.88 nM
compound 7	^1^HA**E**(C8 amine)GTFTSDVSSYLEGQAAKEFIAWLVKGRG^32^	103.96 ± 3.42 nM
compound 8	^1^HA**E**(C12 amine)GTFTSDVSSYLEGQAAKEFIAWLVKGRG^32^	96.73 ± 2.95 nM
compound 9	^1^HA**E**(C16 amine)GTFTSDVSSYLEGQAAKEFIAWLVKGRG^32^	99.74 ± 0.92 nM
compound 10	^1^HAEGTFTS**D**(C8 amine)VSSYLEGQAAKEFIAWLVKGRG^32^	88.35 ± 3.27 nM
compound 11	^1^HAEGTFTS**D**(C12 amine)VSSYLEGQAAKEFIAWLVKGRG^32^	97.35 ± 2.11 nM
compound 12	^1^HAEGTFTS**D**(C16 amine)VSSYLEGQAAKEFIAWLVKGRG^32^	84.57 ± 4.36 nM
compound 13	^1^HAEGTFTSDVSSYL**E**(C8 amine)GQAAKEFIAWLVKGRG^32^	18.74 ± 1.36 nM
compound 14	^1^HAEGTFTSDVSSYL**E**(C12 amine)GQAAKEFIAWLVKGRG^32^	18.43 ± 2.36 nM
compound 15	^1^HAEGTFTSDVSSYL**E**(C16 amine)GQAAKEFIAWLVKGRG^32^	19.74 ± 2.37 nM
compound 16	^1^HAEGTFTSDVSSYLEGQAAK**E**(C8 amine)FIAWLVKGRG^32^	22.31 ± 0.46 nM
compound 17	^1^HAEGTFTSDVSSYLEGQAAK**E**(C12 amine)FIAWLVKGRG^32^	18.73 ± 2.13 nM
compound 18	^1^HAEGTFTSDVSSYLEGQAAK**E**(C16 amine)FIAWLVKGRG^32^	16.54 ± 2.17 nM
compound 19	^1^HAEGTFTSDVSSYLEGQAAKEFIAWLV 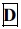 (C8 amine)GRG^32^	19.63 ± 2.14 nM
compound 20	^1^HAEGTFTSDVSSYLEGQAAKEFIAWLV 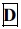 (C12 amine)GRG^32^	20.13 ± 1.97 nM
compound 21	^1^HAEGTFTSDVSSYLEGQAAKEFIAWLV 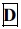 (C16 amine)GRG^32^	17.47 ± 2.75 nM
compound 22	^1^HAEGTFTSDVSSYLEGQAAKEFIAWLV 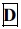 (12 aminododecanoic acid)GRG^32^	21.06 ± 2.34 nM
compound 23	^1^HAEGTFTSDVSSYL**E**(12 aminododecanoic acid)GQAAKEFIAWLVKGRG^32^	24.34 ± 1.96 nM
compound 24	^1^HAEGTFTSDVSSYLEGQAAK**E**(12 aminododecanoic acid)FIAWLVKGRG^32^	17.65 ± 1.76 nM

The sequences of 24 GLP-1 derivatives were listed in Table 1. Fatty acids, fatty amine or amino terminal fatty acid was conjugated with mutant, and conjugating residue was in bold. For compound 19, 20, 21 and 22, Lys residue was replaced with Asp and then conjugated with fatty amine. The binding constants of peptides to GLP-1 receptor were investigated using a cellular ELISA assay; the highlighted GLP-1 derivatives retained similar binding capacities to wild-type GLP-1 and were employed for further investigations in this study. Data represent the mean ± SD, n = 3.
