# Supplementary material for: Variant fatty acid-like molecules Conjugation, novel approaches for extending the stability of therapeutic peptides
Source: Sci Rep. 2015 Dec 11;5:18039. doi: 10.1038/srep18039 (PMC4676015; doi:10.1038/srep18039)
Supplement: Supplementary Information [file srep18039-s1.pdf]

Variant fatty acid-like molecules Conjugation, novel approaches for extending the stability of therapeutic peptides

Ying Li, Yuli Wang, Qunchao Wei, Xuemin Zheng, Lida Tang, Dexin Kong and Min Gong

Supplementary

group	dose (mg/kg/2 days)	Spleen (weight)	thymus gland (weight)
vehicle	--	2.48 ±0.6	2.0 ±0.5
solvent	--	3.52 ±1.8*	2.22 ±0.6
compound 24	5	2.67 ±0.6	2.03 ±0.3
	15	2.94 ±0.6*	2.32 ±0.6
	50	2.91 ±0.8	2.67 ±0.7**

T1. Toxicity studies of compound 24 in Kunming mice.

Legend: Kunming mice (n=30) were treated with compound 24 for 30 days. The doses used in this experiment are 50, 150 and 500 folds of normal dose employed in IPTGG assay (100 μg/kg/2 days), respectively. The data indicated that there is no mortal toxic effect of compound 24 to spleen and thymus gland (*P<0.05, **P<0.01).

group	dose (mg/kg)	ALT	AST	ALP	BUN	CREA	TP	ALB	GLU
vehicle	--	20.74±1.6	108.14±7.2	183.79±16.4	11.50±0.8	48.89±10.8	64.76±1.4	23.55±0.4	3.92±0.3
solvent	--	19.49±2.0	102.39±9.1	143.39±23.6**	11.29±1.4	50.31±9.8	62.83±1.1*	23.11±0.6	4.06±0.8
compound 24	5	22.90±3.4	100.04±10.4	176.31±20.8	9.84±0.9**	48.10±4.3	64.27±2.1	23.64±0.7	4.87±1.0*
	15	22.33±2.9	99.55±12.3	157.87±16.3*	10.28±0.8*	60.18±11.3	64.88±1.1	23.35±0.8	4.06±0.8
	50	25.16±2.6	115.32±11.9	176.0±14.6	10.51±1.4	61.30±13.1	63.75±2.1	23.50±0.8	2.87±0.1**

CONTINUED

group	dose (mg/kg)	WBC	#Neut	#Lymph	#Mono	#Eos	#Baso	#LUC
vehicle	--	1.52 ±0.5	0.25 ±0.1	1.04 ±0.4	0.02 ±0.01	0.21 ±0.1	0.01 ±0.01	0.01 ±0.01
solvent	--	2.68 ±0.7	0.50 ±0.1	2.09 ±0.8	0.03 ±0.02	0.18 ±0.1	0.01 ±0.01	0.01 ±0.01
compound 24	5	1.03 ±0.4*	0.27 ±0.2	0.67 ±0.3	0.01 ±0.01	0.07 ±0.03	0.00 ±0.00	0.01 ±0.01
	15	1.38 ±0.4	0.36 ±0.1	0.90 ±0.3	0.01 ±0.01	0.10 ±0.1	0.00 ±0.00	0.01 ±0.01
	50	1.75 ±0.7	0.36 ±0.1	1.23 ±0.6	0.01 ±0.01	0.14 ±0.1	0.01 ±0.01	0.01 ±0.01

T2. Toxicity study of compound 24 in Wistar rats

Legend: Wistar rats (n=8) were treated with compound 24 for 30 days. The blood samples were collected and analyzed as experimental procedure mentioned previously. The data indicated that there were no toxicologically significant changes in clinical signs, body weight, food and water consumptions and biomedical parameters.
